# Parental awareness and practices regarding paracetamol use in children: A cross-sectional study from Pakistan

**DOI:** 10.1371/journal.pgph.0005358

**Published:** 2025-10-23

**Authors:** Muhammad Saad Nadeem Butt, Sana Shah

**Affiliations:** 1 Islam Medical College, Sialkot, Pakistan; 2 Department of Medical Education, Islam Medical College, Sialkot, Pakistan; PLOS: Public Library of Science, UNITED STATES OF AMERICA

## Abstract

Paracetamol is the one of the most widely administered drug as an analgesic and antipyretic due to its efficacy, safety, and over-the-counter (OTC) nature and is present in various pharmaceutical forms. Paracetamol usage in children is a global concern yet understudied in Pakistan. The present study was conducted to assess parental awareness regarding paracetamol usage and toxicity, highlighting critical public health risks and informing policy interventions aimed at lowering medication-associated toxicity in low-resource settings. This cross-sectional study aimed at assessing parental awareness in Sialkot city of Pakistan via a Urdu-language questionnaire (adapted from previous literature after pilot testing and expert review) was used as a tool to collect data. Questionnaire was distributed in six pediatric healthcare facilities. 450 parents were approached out of which only 420 gave consent and met inclusion and exclusion criteria. 2 responses were removed due to missing data. A total of 418 parents were interviewed, of which 67 % were mothers. Almost 99 % used it as an antipyretic, 18 % used it as an analgesic, and 81 %(n = 340) of the participants employed its use for the symptoms of illness (cough, flu, and vomiting). Most participants used paracetamol as a syrup. Health-care providers were the primary source of knowledge for paracetamol dosage. Only 32.8 % of participants were aware that a paracetamol overdose can cause harm. Approximately 75.8 % of the 418 participants scored below 66.67 % (4/6 questions) on the knowledge score and are considered to have insufficient knowledge. The current study highlights the lack of knowledge in parents regarding proper paracetamol usage which poses a significant risk of paracetamol poisoning. It underscores the importance of implementation of educational initiatives aimed at reducing the risks of toxicity and increasing awareness and knowledge regarding paracetamol among parents.

## Introduction

### Background

Paracetamol, also known as acetaminophen, is present in various pharmaceutical forms, including drops, syrups, and tablets, and is often combined with other medications. It is considered one of the most efficacious and safe over-the-counter (OTC) drugs for pain and fever management in both children and adults [1,2]. Despite its safety and efficacy, its improper use (overdose and underdose) can lead to serious health effects. Overdosing can result in hepatic failure, which is a leading cause of pediatric poisoning worldwide [3,4], whereas underdosing may lead to improper fever management, prompting the further use of other antipyretics or antibiotics.

Paracetamol toxicity is one of the most common causes of toxicity in many countries [5,6] including in the USA where the paracetamol toxicity continues to be a serious health concern [7] A study in India revealed that paracetamol poisoning accounts for 21% of acute liver failure cases in the paediatrics population [4].

Several studies have reported that parents often lack knowledge regarding paracetamol dosage, frequency, and possible side effects when it is used in children [8–12]. A study in Palestine revealed that 95% of parents lacked knowledge regarding paracetamol usage in children [8]. Similarly, a study in Sri Lanka showed that 43% of parents administer a high dose of paracetamol to children [13].

This lack of knowledge is especially concerning in developing countries, where access to healthcare and education is limited. Owing to its OTC nature, widespread availability, and general presence in homes, paracetamol is often administered without medical consultation, which may lead to medication errors [14,15]. Furthermore, some parents combine several OTC products containing paracetamol to combat fever, resulting in cumulative poisoning. Many parents believe that paracetamol is safe for use at any dosage, further increasing the risk of misuse. A study in Turkey revealed that these misconceptions led to increased emergency room visits for drug-related complications [16]. This problem is amplified in developing countries where healthcare facilities are already stretched thin and medication awareness is limited [17,18].

Several international studies in regions such as Southeast Asia, Africa, and the Middle East, have highlighted the importance of parental education in reducing medication errors [9,10,19–24]. A study in Australia revealed that parents incorrectly administered paracetamol to their children, often giving higher-than-recommended doses, which leads to severe poisoning [25].

Owing to its OTC nature, it is often self-medicated in Pakistan, especially among children. Despite its widespread usage, studies assessing parental knowledge, dosing practices and toxicity awareness are scarce.

In Pakistan, despite paracetamol’s widespread use, there is a scarce of studies on parental knowledge and awareness regarding toxicity and dosing practices. In contrast, existing studies from Palestine and Saudi Arabia highlights an alarming rate of misuse [8,9].

This is the first cross-sectional study in Pakistan to assess knowledge and awareness regarding its usage and provides valuable insights aimed at lowering paracetamol misuse.

### Objective

This study aims to identify knowledge gaps in parental awareness of paracetamol usage and aims to informs relevant authorities to take significant measures for future interventions aimed at reducing the risk of paracetamol toxicity in children.

## Methods

### Study design and settings

This cross-sectional study was conducted in six paediatric healthcare facilities in Sialkot over a span of six months from 13September 2024 – 28 February 2025. These selected healthcare facilities serve both rural and urban populations.

### Inclusion and exclusion criteria

#### Inclusion.

- Children ≤ 8 years were selected as they rely on parents for medicine administration.

#### Exclusion.

- Parents of children suffering from any chronic illness, mental disorders or congenital malformations were excluded.
- The participants were required to provide informed consent. Those who did not provide informed consent were not included.[26]

### Sampling strategy

Parents were recruited via convenience sampling during routine paediatric visits. Parents were approached in waiting areas, explained the study objectives and checked for eligibility. Among the 450 parents approached, 420 agreed to participate and were eligible and consented. Of these, 2 responses were excluded because they were incomplete.

### Sample size and sampling techniques

To calculate the sample size, we used Cochran’s formula [26] as follows:


$$𝐍={𝐙}^{2}𝐩\left(1-𝐩\right)/{𝐞}^{2}$$

where z is the z value (z score), i.e., the desired confidence level, which we have taken as 95%, so Z = 1.96, whereas e is the desired level of precision (margin of error is kept at 0.05 for ±5%).

P is the assumed proportion of the population, which is taken as **0.5** to ensure maximized variability due to a lack of prior Pakistan data.

The required number of samples to be collected was **385**. To ensure the reliability of the study, **417** samples were collected.

### Questionnaire development and validation

The questionnaire was adapted from another study in Palestine [4]. The key steps for modifying the questionnaire included the following steps:

- Translation: Forward-translated to Urdu by bilingual experts and then back-translated to English to ensure semantic equivalence.
- Pilot testing: Twenty parents (excluded from the final sample) were included. Cronbach’s α = 0.78 indicated good internal consistency for the exploratory studies.[27]
- Cultural Adaptation: Reviewed by a panel of pediatricians and pharmacists to align with local practices (e.g., replacing brands with more common brands in Pakistan).

The final tool included the following:

- Section 1: Socio demographics (age, education, number of children).
- Section 2: Paracetamol use (indications, dosage forms, sources of information).
- Section 3: Toxicity awareness (overdose risks, organ damage knowledge)

At the end of the interview, parents were given an educational orientation regarding their paracetamol dosage and toxicity.

### Data collection protocol

Trained interviewers conducted face-to-face interviews in Urdu. To minimize bias:

- The participants were assured of anonymity.
- The responses were cross-verified in real time (e.g., asking parents to demonstrate dose measurements).
- Incomplete questionnaires (<2%) were excluded post hoc.

### Statistical analysis

The data were analysed via SPSS v.26.

1. Descriptive statistics: Frequencies, percentages
2. Knowledge scoring: knowledge score of 4 out of 6 (66.7%) was used as a cutoff to indicate limited knowledge. This threshold was adapted from previous literature [8] that used similar classification to assess medication knowledge. However, it is not a validated standard and was used here only for descriptive analysis. Although knowledge scores are continuous by nature, a dichotomized approach was applied for descriptive clarity

### Ethical approval and consent to participate

This study was performed in accordance with the Declaration of Helsinki. Ethical approval was obtained from the institutional review board of Islam Medical College (IRB approval number 900/IMC/ERC/000103). Responses were recorded only after written informed consent was obtained from the participants.

## Results

### Sociodemographic history of the children and parents

417 parents with children aged eight years or younger participated, with the majority being mothers (67%). With respect to educational background, 92.8% of the participants held a bachelor’s degree, 5% had a high school diploma, and 2.2% had completed middle school. Among the children presenting with parents, almost half (50.7%) were male, and the remaining 49.3% were female, which provided a balanced view of parental knowledge and behaviour. The demographic data of the parents and their children are given in Table 1 (also shown in the S1 File).

**Table 1:** Sociodemographic history of the children and parents.

Variable	Frequency (n=417)
**Gender**	
Male	212
Female	205
**Child: Age (Years)**	
1	6
2	3
3	186
4	82
5	35
6	45
7	44
8	16
**Hospital admission history**	
Yes	84
No	333
**Relationship of Parent**	
Father	137
Mother	280
**Age of the parent (In years)**	
20-25	3
25-30	93
30-35	307
35-40	12
40-45	2
**Education level of the parent**	
Middle school	9
High school	21
Bachelor	387
**Number of children under 8 years**	
1	159
2	175
3	72
4	12

### Awareness regarding paracetamol products and indications

#### Self-medication.

With respect to paracetamol usage without consultation, 68.4% of parents reported giving their children paracetamol without prior consultation. Furthermore, many parents were unaware of the generic name paracetamol and instead recognized only the brand names (Table 2).

**Table 2:** Awareness regarding paracetamol products among the participants.

Used paracetamol for the child without consulting a doctor	
Yes	286
No	7
Don’t know what paracetamol is	124
**Used any of the following products (containing paracetamol) for the child**	
Panadol	389
Calpol + Panadol	28
**Dosage form of paracetamol used**	
Tablet	0
Syrup	411
Suppository	0
Drops	6

**Indications (**Table 3**):** The reasons for use are summarized in Table 3. Almost 99% of parents used paracetamol as an antipyretic, whereas 18% of parents also used paracetamol as an analgesic. A small proportion of the population uses paracetamol for sedation. In addition, the majority of parents used it for the treatment of symptoms of illness (cough, flu and vomiting).

**Table 3:** Paracetamol indications as perceived by the participating parents (Note: Participants could select multiple indications so that the total score exceeds 418 because of multiple responses).

Paracetamol used for	Frequency
Antipyretic	413
Analgesic	75
Symptoms of illness (cough, flu and vomiting)	340
Sedation	27

#### Toxicity awareness.

Parents showed limited awareness, as 45% of participants were unaware of the harm caused by an overdose, whereas only 32.8% were aware that an overdose can cause harm to the body, and 22.2% were unaware of any harm caused by its overdose (Table 4). In terms of harm caused by an overdose, 40.9% believed that its toxicity can cause stomach damage, followed by 32.3% for liver damage and 24.2% for immunosuppression, and only 1.2% believed that it can cause renal damage.

**Table 4:** Participating parents’ responses to questions regarding knowledge of paracetamol use.

Variable	Frequency
**Temperature threshold for administering paracetamol**	
99°F (37.2 C)	17
100°F (37.7 C)	400
**Daily maximum frequency of paracetamol**	
One dose	2
Two dose	261
Three dose	153
Four dose	1
**Time allowed between two doses**	
Less than 4 hour	16
4–6 hour	367
More than 6 hour	32
Don’t know	2
**Time allowed for use after opening a syrup bottle**	
3 months	3
6 months	3
Until expiry	411
**Paracetamol overdose can cause serious side effects**	
Yes	137
No	93
Maybe	187
**The type of damage paracetamol overdose causes**	
Renal failure	5
Liver damage	135
Stomach damage	171
Immunosuppressant	101
Other	5
**Knowledge score**	
2	20
3	132
4	164
5	95
6	6

#### Dosage understanding.

In terms of dosage understanding, 36.6% reported that the maximum number of doses should be three, whereas 62.4% believed that it should be two.

Furthermore, 87.8% of parents reported time between two consecutive doses is 4–6 hours.

Most parents (98.6%) reported using paracetamol syrup until the expiry date, whereas only 1.4% stopped using it after 3 or 6 months.

When the participants were questioned about the factors influencing dosage, child weight (38.3%) was the most common factor influencing dose, which was closely followed by the severity of illness (31.6%), and 28.7% of the participants reported basing dosage on previous use. A total of 93.8% of the participants believed that the number of doses depended upon the severity of the illness.

Many parents prefer syrup as the primary dosage form because of its ease of use. A total of 94.5% of parents relied on previous experience for dosing information, whereas only 0.7% referred to a doctor.

When questioned about temperature, approximately 95.7% indicated 100°F (37.7°C) for use. Knowledge among participants regarding paracetamol dosage and toxicity is measured by a knowledge score of 0--6, with 75.8% of the population having a knowledge score of ≤4, which indicates insufficient knowledge among parents regarding paracetamol use, suggesting that a significant number of the paediatric population is at risk of toxicity.

### Indications for paracetamol usage

In the present study, 99% of the participants used paracetamol as an antipyretic, 18% used it for analgesia, and 82.2% used it for symptoms of illness (cough, flu and vomiting).

### Response regarding paracetamol related practice

A total of 98.3% of parents preferred the oral route, which is also recommended because of its precision in estimating the dose. A total of 2.9% of parents stated that convenience is the reason they prefer the oral route, while 1.4% reported its efficacy, and 92.3% reported doctors’ advice behind its oral use.

According to our study, most parents (95%) use incorrect measuring tools such as tablespoons or teaspoons. This finding indicates that kitchen utensils are still used for measuring doses, which leads to higher rates of toxic dose administration in children. Only approximately 3.3% used correct measuring tools (Table 5). Only 32.8% of the participants in the study knew that paracetamol can be toxic at high doses, while 22% believed that paracetamol is safe regardless of its dose, and 45% were not sure about its potential harm to the body. In addition, 40.9% of parents believed that paracetamol caused stomach damage, whereas 24.2% believed that it caused immunosuppression. Only 32.3% correctly believed that it caused liver damage, and 1.2% believed that it caused renal damage.

**Table 5:** Participating parents’ responses to questions on Paracetamol-related practices.

Variable	Frequency
**Paracetamol dosage form preferred**	
Syrup	411
Suppository	0
Drops	6
Tablet	0
**Syrup measuring tool used**	
Teaspoon	60
Tablespoon	337
Measuring cap	14
Haven’t used it previously	6
**Reason for choosing this dosage form**	
Price	
Efficacy	6
Easy to administer	12
Doctor’s recommendation	386
Pharmacist recommendation	13
**Reason for paracetamol used without prescription**	
No need to visit the doctor	2
High consultation cost	1
Previous experience with the same symptom	410
Other	4
**Reason for repeating the dose**	
Severity of illness	392
Age	15
Weight	3
Doctor’s consultation	7
**Source of information depends upon**	
Doctor’s consultation	395
Pharmacist consultation	3
Own knowledge	2
Relative and friends	1
Experience from previous use	14
Medication leaflet	2

When parents were asked about dosing intervals and daily dosing, 87.8% of parents believed that the dosing interval was 4–6 hours. A total of 62.4% believed that daily dosing is performed twice a day, and 36.6% believed that it is performed thrice a day, which shows that parents are well aware of the dosing interval. A total of 38.3% of the population believed that weight is a factor that influences dosing quantity, and 28.7% believed that dose was measured by age. A total of 31.6% incorrectly believed that the severity of illness was associated with the dosing quantity, which increases the risk of toxicity.

## Discussion

Our findings in the present study raises concern about significant knowledge gaps and potential for misuse in the community.

About 68.4% of parents reported administering paracetamol to their children without consulting healthcare, which is consistent with international studies [8,9,12,13]

Our study found that only 32.8% of respondents were aware that a paracetamol overdose could cause serious harm, with 40.9% of participants associate the toxicity with stomach damage and only 32.3% recognizing liver damage as the primary risk. This mirrors findings from previous studies in Turkey and Sri Lanka, where misconceptions about the toxic effects of paracetamol were widespread [13,16]. The lack of awareness regarding hepatic toxicity is alarming, given that paracetamol overdose is a leading cause of acute liver failure in both children and adults globally [3,4,5].

Only 3.3% reported using measuring cup for measuring paracetamol(syrup) while majority relying on household utensils like tablespoon and teaspoons which is the major cause of unintentional overdosing in children [10,22,25,28]. This may also leads to sub therapeutic dosing leading to usage of others drugs [25,28]

Despite widespread use, knowledge of dosing intervals and dose frequency was inconsistent among participants. While 87.8% identified the appropriate dosing interval (4–6 hours), only 36.6% identified the three doses as daily dosing frequency. This discrepancy between partial and complete knowledge has also been highlighted in Australian and African studies, indicating a global trend in parental underestimation of dosing precision [11,17,29].

Sociodemographic data of participants indicates that 92.8% of parents in present study held a bachelor degree or higher but knowledge regarding paracetamol dosage and toxicity is limited, highlighting that formal education does not necessarily equate to medication literacy which emphasizes the need of targeted public health interventions [19,20].

94.5% of participants based their dosage on past experience and only 0.7% rely on doctor’s advice. This over reliance on experiential knowledge increases the chances of cumulative poisoning when multiple paracetamol containing products are administered concurrently, as observed in global poisoning surveillance reports [7,30,23]

These findings aligns with the WHO “Medication without harm” campaign which identifies OTC misuse is a key focus on reducing medication toxicity [31]

International studies also support the effectiveness of caregiver-targeted interventions in reducing medication errors [21,22] These findings highlight the importance of interventions to improve awareness and parental knowledge regarding paracetamol usage in children to avoid the risk of toxicity. Following policy implications can be used which aligns with WHO recommendation and international experience

- OTC Regulation: Restricting paracetamol sales to pharmacies, requiring counselling for caregivers
- Standardized tools: Government-funded distribution dosing charts and correct measuring tools
- Educational Campaigns: Collaborate with community health workers to deliver workshops in rural and urban areas.

### Strengths and limitations

#### Strengths.

1. A large sample size increases the study’s efficacy.
2. To ensure complete forms and reduce bias, face‒to‒face interviews were conducted, and the responses were verified in real time.
3. Rigorous questionnaire validation ensures cultural relevance.

#### Limitations.

The following are some limitations of the study.

1. Selection bias: Clinical-based interviews exclude those that avoid healthcare and urban based healthcare facilities may have attracted more educated individuals
2. Social desirability bias: Parents may overstate adherence to “ideal” practices or hesitancy in sharing medical information, which may be due to fear of judgement or lack of awareness.
3. Using an arbitrary cutoff to classify knowledge may oversimplify the range of understanding. Treating the score as continuous in future studies could yield more nuanced insights
4. Recall bias: Difficulty in recalling past information.
5. Convenience sampling from healthcare institutions may inadequately represent rural and low-literacy groups, hence constraining generalizability. Therefore, future studies should adopt stratified sampling to ensure generalizability.
6. As 92% of participants held a bachelor’s degree, the sample may not represent the general Pakistani population, limiting external validity.

## Conclusion

The current study highlights significant knowledge gaps regarding paracetamol dose quantity, interval between two doses, toxicity and number of doses daily. Misconceptions that paracetamol is always safe may increase the risk of toxicity, underscoring the need for targeted public health awareness initiatives and future studies.

## Supporting information

S1 File SPSS output supporting the study findings. (DOCX)

S2 File Raw Excel dataset used for analysis in the study. (XLSX)
