# Supplementary material for: Parental awareness and practices regarding paracetamol use in children: A cross-sectional study from Pakistan
Source: PLOS Glob Public Health. 2025 Oct 23;5(10):e0005358. doi: 10.1371/journal.pgph.0005358 (PMC12548860; doi:10.1371/journal.pgph.0005358)
Supplement: S1 File — (DOCX) [file pgph.0005358.s001.docx]

FREQUENCIES VARIABLES=ParentGender ParentAge ParentEducation ChildGender ChildAge Hospit alAdmission

UsedWithoutConsulting DosageForm Antipyretic Analgesic SymptomsofIllness Sedation Te mpThreshold

MaxDosesPerDay IntervalBetweenDoses UseAfterOpen OverdoseAwareness DamageType Measur eTool BrandUsed

ReasonforNoRx InfoSource ReasonforRepeatDose DosageFormReason Knowledge_score

Knowledge_score_recoded /ORDER=ANALYSIS.

# Frequencies

**Notes**

| Output Created |  | 28-JUL-2025 19:14:16 |
| --- | --- | --- |
| Comments |  |  |
| Input | Active Dataset | DataSet2 |
|  | Filter | <none> |
|  | Weight | <none> |
|  | Split File | <none> |
|  | N of Rows in Working Data  File | 417 |
| Missing Value Handling | Definition of Missing | User-defined missing values are treated as missing. |
|  | Cases Used | Statistics are based on all cases with valid data. |

**Notes**

| Syntax |  | FREQUENCIES  VARIABLES=ParentGend er ParentAge  ParentEducation  ChildGender ChildAge  HospitalAdmission  UsedWithoutConsulting  DosageForm Antipyretic  Analgesic  SymptomsofIllness  Sedation TempThreshold  MaxDosesPerDay  IntervalBetweenDoses  UseAfterOpen  OverdoseAwareness  DamageType  MeasureTool BrandUsed  ReasonforNoRx  InfoSource  ReasonforRepeatDose  DosageFormReason  Knowledge_score    Knowledge_score_recode d  /ORDER=ANALYSIS. |
| --- | --- | --- |
| Resources | Processor Time | 00:00:00.02 |
|  | Elapsed Time | 00:00:00.02 |

**Statistics**

|  |  | Parent Gender | Parent Age | Parent Education | Child Gender | Child Age |
| --- | --- | --- | --- | --- | --- | --- |
| N | Valid | 417 | 417 | 417 | 417 | 417 |
|  | Missing | 0 | 0 | 0 | 0 | 0 |

**Statistics**

|  |  | Hospital  Admission | Used Without  Consulting | Dosage Form | Antipyretic | Analgesic |
| --- | --- | --- | --- | --- | --- | --- |
| N | Valid | 417 | 417 | 417 | 417 | 417 |
|  | Missing | 0 | 0 | 0 | 0 | 0 |

**Statistics**

|  |  | Symptoms of  Illness | Sedation | Temp Threshold | Max Doses Per  Day | Interval  Between Doses |
| --- | --- | --- | --- | --- | --- | --- |
| N | Valid | 417 | 417 | 417 | 417 | 417 |
|  | Missing | 0 | 0 | 0 | 0 | 0 |

**Statistics**

|  |  | Use After Open | Overdose Awareness | Damage Type | Measure Tool | Brand Used |
| --- | --- | --- | --- | --- | --- | --- |
| N | Valid | 417 | 417 | 417 | 417 | 417 |
|  | Missing | 0 | 0 | 0 | 0 | 0 |

**Statistics**

|  |  | Reason for No  Rx | Info Source | Reason for Repeat Dose | Dosage Form  Reason | Knowledge_sco re |
| --- | --- | --- | --- | --- | --- | --- |
| N | Valid | 417 | 417 | 417 | 417 | 417 |
|  | Missing | 0 | 0 | 0 | 0 | 0 |

**Statistics**

Knowledge_sco

re_recoded

N

Valid

Missing

417

0

# Frequency Table

**Parent Gender**

|  |  | Frequency | Percent | Valid Percent | Cumulative  Percent |
| --- | --- | --- | --- | --- | --- |
| Valid | Father | 137 | 32.9 | 32.9 | 32.9 |
|  | Mother | 280 | 67.1 | 67.1 | 100.0 |
|  | Total | 417 | 100.0 | 100.0 |  |

**Parent Age**

|  | Frequency | | Percent | Valid Percent | Cumulative  Percent |
| --- | --- | --- | --- | --- | --- |
| Valid | 20 | 3 | .7 | .7 | .7 |
|  | 25 | 93 | 22.3 | 22.3 | 23.0 |
|  | 30 | 307 | 73.6 | 73.6 | 96.6 |
|  | 35 | 12 | 2.9 | 2.9 | 99.5 |
|  | 40 | 2 | .5 | .5 | 100.0 |
|  | Total | 417 | 100.0 | 100.0 |  |

**Parent Education**

|  |  | Frequency | Percent | Valid Percent | Cumulative  Percent |
| --- | --- | --- | --- | --- | --- |
| Valid | Bachelor | 387 | 92.8 | 92.8 | 92.8 |
|  | High school | 21 | 5.0 | 5.0 | 97.8 |
|  | Middle school | 9 | 2.2 | 2.2 | 100.0 |
|  | Total | 417 | 100.0 | 100.0 |  |

**Child Gender**

|  |  | Frequency | Percent | Valid Percent | Cumulative  Percent |
| --- | --- | --- | --- | --- | --- |
| Valid | Female | 205 | 49.2 | 49.2 | 49.2 |
|  | Male | 212 | 50.8 | 50.8 | 100.0 |
|  | Total | 417 | 100.0 | 100.0 |  |

**Child Age**

|  |  | Frequency | Percent | Valid Percent | Cumulative  Percent |
| --- | --- | --- | --- | --- | --- |
| Valid | 1 | 6 | 1.4 | 1.4 | 1.4 |
|  | 2 | 3 | .7 | .7 | 2.2 |
|  | 3 | 186 | 44.6 | 44.6 | 46.8 |
|  | 4 | 82 | 19.7 | 19.7 | 66.4 |
|  | 5 | 35 | 8.4 | 8.4 | 74.8 |
|  | 6 | 45 | 10.8 | 10.8 | 85.6 |
|  | 7 | 44 | 10.6 | 10.6 | 96.2 |
|  | 8 | 16 | 3.8 | 3.8 | 100.0 |
|  | Total | 417 | 100.0 | 100.0 |  |

**Hospital Admission**

|  | Frequency | | Percent | Valid Percent | Cumulative  Percent |
| --- | --- | --- | --- | --- | --- |
| Valid | No | 333 | 79.9 | 79.9 | 79.9 |
|  | Yes | 84 | 20.1 | 20.1 | 100.0 |
|  | Total | 417 | 100.0 | 100.0 |  |

**Used Without Consulting**

|  |  | Frequency | Percent | Valid Percent | Cumulative  Percent |
| --- | --- | --- | --- | --- | --- |
| Valid | Don’t know | 124 | 29.7 | 29.7 | 29.7 |
|  | No | 7 | 1.7 | 1.7 | 31.4 |
|  | Yes | 286 | 68.6 | 68.6 | 100.0 |
|  | Total | 417 | 100.0 | 100.0 |  |

**Dosage Form**

|  |  | Frequency | Percent | Valid Percent | Cumulative  Percent |
| --- | --- | --- | --- | --- | --- |
| Valid | Drops | 6 | 1.4 | 1.4 | 1.4 |
|  | Syrup | 411 | 98.6 | 98.6 | 100.0 |
|  | Total | 417 | 100.0 | 100.0 |  |

**Antipyretic**

|  |  | Frequency | Percent | Valid Percent | Cumulative  Percent |
| --- | --- | --- | --- | --- | --- |
| Valid | No | 4 | 1.0 | 1.0 | 1.0 |
|  | Yes | 413 | 99.0 | 99.0 | 100.0 |
|  | Total | 417 | 100.0 | 100.0 |  |

**Analgesic**

|  |  | Frequency | Percent | Valid Percent | Cumulative  Percent |
| --- | --- | --- | --- | --- | --- |
| Valid | No | 342 | 82.0 | 82.0 | 82.0 |
|  | Yes | 75 | 18.0 | 18.0 | 100.0 |
|  | Total | 417 | 100.0 | 100.0 |  |

**Symptoms of Illness**

|  |  | Frequency | Percent | Valid Percent | Cumulative  Percent |
| --- | --- | --- | --- | --- | --- |
| Valid | No | 77 | 18.5 | 18.5 | 18.5 |
|  | Yes | 340 | 81.5 | 81.5 | 100.0 |
|  | Total | 417 | 100.0 | 100.0 |  |

**Sedation**

|  |  | Frequency | Percent | Valid Percent | Cumulative  Percent |
| --- | --- | --- | --- | --- | --- |
| Valid | No | 390 | 93.5 | 93.5 | 93.5 |
|  | Yes | 27 | 6.5 | 6.5 | 100.0 |
|  | Total | 417 | 100.0 | 100.0 |  |

|  |  | Frequency | Percent | Valid Percent | Cumulative  Percent |
| --- | --- | --- | --- | --- | --- |
| Valid | <4h | 16 | 3.8 | 3.8 | 3.8 |
|  | >6h | 32 | 7.7 | 7.7 | 11.5 |
|  | 4-6h | 367 | 88.0 | 88.0 | 99.5 |
|  | Don’t know | 2 | .5 | .5 | 100.0 |
|  | Total | 417 | 100.0 | 100.0 |  |

**Temp Threshold**

|  |  | Frequency | Percent | Valid Percent | Cumulative  Percent |
| --- | --- | --- | --- | --- | --- |
| Valid | 99 | 17 | 4.1 | 4.1 | 4.1 |
|  | 100 | 400 | 95.9 | 95.9 | 100.0 |
|  | Total | 417 | 100.0 | 100.0 |  |

**Max Doses Per Day**

|  |  | Frequency | Percent | Valid Percent | Cumulative  Percent |
| --- | --- | --- | --- | --- | --- |
| Valid | 1 | 2 | .5 | .5 | .5 |
|  | 2 | 261 | 62.6 | 62.6 | 63.1 |
|  | 3 | 153 | 36.7 | 36.7 | 99.8 |
|  | 4 | 1 | .2 | .2 | 100.0 |
|  | Total | 417 | 100.0 | 100.0 |  |

**Interval Between Doses Use After Open**

|  |  | Frequency | Percent | Valid Percent | Cumulative  Percent |
| --- | --- | --- | --- | --- | --- |
| Valid | 3 months | 3 | .7 | .7 | .7 |
|  | 6 months | 3 | .7 | .7 | 1.4 |
|  | Until expiry | 411 | 98.6 | 98.6 | 100.0 |
|  | Total | 417 | 100.0 | 100.0 |  |

**Overdose Awareness**

|  |  | Frequency | Percent | Valid Percent | Cumulative  Percent |
| --- | --- | --- | --- | --- | --- |
| Valid | Maybe | 187 | 44.8 | 44.8 | 44.8 |
|  | No | 93 | 22.3 | 22.3 | 67.1 |
|  | Yes | 137 | 32.9 | 32.9 | 100.0 |
|  | Total | 417 | 100.0 | 100.0 |  |

**Damage Type**

|  |  | Frequency | Percent | Valid Percent | Cumulative  Percent |
| --- | --- | --- | --- | --- | --- |
| Valid | Immunosuppression | 101 | 24.2 | 24.2 | 24.2 |
|  | Liver | 135 | 32.4 | 32.4 | 56.6 |
|  | Other | 5 | 1.2 | 1.2 | 57.8 |
|  | Renal | 5 | 1.2 | 1.2 | 59.0 |
|  | Stomach | 171 | 41.0 | 41.0 | 100.0 |
|  | Total | 417 | 100.0 | 100.0 |  |

**Measure Tool**

|  |  | Frequency | Percent | Valid Percent | Cumulative  Percent |
| --- | --- | --- | --- | --- | --- |
| Valid | Cap | 14 | 3.4 | 3.4 | 3.4 |
|  | Not Used | 6 | 1.4 | 1.4 | 4.8 |
|  | Tablespoon | 337 | 80.8 | 80.8 | 85.6 |
|  | Teaspoon | 60 | 14.4 | 14.4 | 100.0 |
|  | Total | 417 | 100.0 | 100.0 |  |

**Brand Used**

|  | Frequency | | Percent | Valid Percent | Cumulative  Percent |
| --- | --- | --- | --- | --- | --- |
| Valid | Calpol + Panadol | 28 | 6.7 | 6.7 | 6.7 |
|  | Panadol | 389 | 93.3 | 93.3 | 100.0 |
|  | Total | 417 | 100.0 | 100.0 |  |

**Reason for No Rx**

|  |  | Frequency | Percent | Valid Percent | Cumulative  Percent |
| --- | --- | --- | --- | --- | --- |
| Valid | Doctor fee too expensive | 1 | .2 | .2 | .2 |
|  | No need to visit the doctor | 2 | .5 | .5 | .7 |
|  | Other | 4 | 1.0 | 1.0 | 1.7 |
|  | Prior experience | 410 | 98.3 | 98.3 | 100.0 |
|  | Total | 417 | 100.0 | 100.0 |  |

**Info Source**

|  |  | Frequency | Percent | Valid Percent | Cumulative  Percent |
| --- | --- | --- | --- | --- | --- |
| Valid | Doctor | 395 | 94.7 | 94.7 | 94.7 |
|  | Medication leaflet | 2 | .5 | .5 | 95.2 |
|  | Own knowledge | 2 | .5 | .5 | 95.7 |
|  | Pharmacist | 3 | .7 | .7 | 96.4 |
|  | Previous experience | 14 | 3.4 | 3.4 | 99.8 |
|  | Relatives/Friends | 1 | .2 | .2 | 100.0 |
|  | Total | 417 | 100.0 | 100.0 |  |

**Reason for Repeat Dose**

|  |  | Frequency | Percent | Valid Percent | Cumulative  Percent |
| --- | --- | --- | --- | --- | --- |
| Valid | Age | 15 | 3.6 | 3.6 | 3.6 |
|  | Doctor consultation | 7 | 1.7 | 1.7 | 5.3 |
|  | Severity of illness | 392 | 94.0 | 94.0 | 99.3 |
|  | Weight | 3 | .7 | .7 | 100.0 |
|  | Total | 417 | 100.0 | 100.0 |  |

**Dosage Form Reason**

|  |  | Frequency | Percent | Valid Percent | Cumulative  Percent |
| --- | --- | --- | --- | --- | --- |
| Valid | Doctor recommendation | 386 | 92.6 | 92.6 | 92.6 |
|  | Easy to use | 12 | 2.9 | 2.9 | 95.4 |
|  | Efficacy | 6 | 1.4 | 1.4 | 96.9 |
|  | Pharmacist recommendation | 13 | 3.1 | 3.1 | 100.0 |
|  | Total | 417 | 100.0 | 100.0 |  |

**Knowledge_score**

|  |  | Frequency | Percent | Valid Percent | Cumulative  Percent |
| --- | --- | --- | --- | --- | --- |
| Valid | 2.00 | 20 | 4.8 | 4.8 | 4.8 |
|  | 3.00 | 132 | 31.7 | 31.7 | 36.5 |
|  | 4.00 | 164 | 39.3 | 39.3 | 75.8 |
|  | 5.00 | 95 | 22.8 | 22.8 | 98.6 |
|  | 6.00 | 6 | 1.4 | 1.4 | 100.0 |
|  | Total | 417 | 100.0 | 100.0 |  |

**Knowledge_score_recoded**

|  |  | Frequency | Percent | Valid Percent | Cumulative  Percent |
| --- | --- | --- | --- | --- | --- |
| Valid | Insufficent | 316 | 75.8 | 75.8 | 75.8 |
|  | Sufficent | 101 | 24.2 | 24.2 | 100.0 |
|  | Total | 417 | 100.0 | 100.0 |  |

DESCRIPTIVES VARIABLES=Knowledge_score /STATISTICS=MEAN STDDEV MIN MAX.

# Descriptives

**Notes**

| Output Created |  | 28-JUL-2025 19:14:40 |
| --- | --- | --- |
| Comments |  |  |
| Input | Active Dataset | DataSet2 |
|  | Filter | <none> |
|  | Weight | <none> |
|  | Split File | <none> |
|  | N of Rows in Working Data  File | 417 |
| Missing Value Handling | Definition of Missing | User defined missing values are treated as missing. |
|  | Cases Used | All non-missing data are used. |
| Syntax |  | DESCRIPTIVES  VARIABLES=Knowledge_ score  /STATISTICS=MEAN STDDEV MIN MAX. |
| Resources | Processor Time | 00:00:00.00 |
|  | Elapsed Time | 00:00:00.02 |

**Descriptive Statistics**

|  | N | Minimum | Maximum | Mean | Std. Deviation |
| --- | --- | --- | --- | --- | --- |
| Knowledge_score | 417 | 2.00 | 6.00 | 3.8441 | .87825 |
| Valid N (listwise) | 417 |  |  |  |  |

Page
